# Functional and evolutionary study of *MLO* gene family in the regulation of *Sclerotinia* stem rot resistance in *Brassica napus* L.

**DOI:** 10.1186/s13068-023-02325-z

**Published:** 2023-05-23

**Authors:** Jie Liu, Yupo Wu, Xiong Zhang, Rafaqat Ali Gill, Ming Hu, Zetao Bai, Chuanji Zhao, Yi Zhang, Yueying Liu, Qiong Hu, Xiaohui Cheng, Junyan Huang, Lijiang Liu, Shunping Yan, Shengyi Liu

**Affiliations:** 1 The Key Laboratory of Biology and Genetic Improvement of Oil Crops, The Ministry of Agriculture and Rural Affairs of the PRC, Oil Crops Research Institute, Chinese Academy of Agricultural Sciences, Wuhan, 430062 People’s Republic of China; 2 College of Life Science and Technology, Center of Integrative Biology, Interdisciplinary Science Research Institute, Huazhong Agricultural University, Wuhan, 430070 China; 3 Hubei Hongshan Laboratory, Wuhan, 430070 China

**Keywords:** *Brassica napus* L., Genome-wide association studies, Sclerotinia stem rot, *MLO*, Evolution, Transcriptome, Gene expression

## Abstract

**Background:**

Oilseed rape (*Brassica napus* L.) is known as one of the most important oilseed crops cultivated around the world. However, its production continuously faces a huge challenge of Sclerotinia stem rot (SSR), a destructive disease caused by the fungus *Sclerotinia sclerotiorum*, resulting in huge yield loss annually. The SSR resistance in *B. napus* is quantitative and controlled by a set of minor genes. Identification of these genes and pyramiding them into a variety are a major strategy for SSR resistance breeding in *B. napus*.

**Results:**

Here, we performed a genome-wide association study (GWAS) using a natural population of *B. napus* consisting of 222 accessions to identify *BnaA08g25340D* (*BnMLO2_2*) as a candidate gene that regulates the SSR resistance. *BnMLO2_2* was a member of seven homolog genes of *Arabidopsis Mildew Locus O 2* (*MLO2*) and the significantly SNPs were mainly distributed in the promoter of *BnMLO2_2*, suggesting a role of *BnMLO2_2* expression level in the regulation of SSR resistance. We expressed *BnMLO2_2* in Arabidopsis and the transgenic plants displayed an enhanced SSR resistance. Transcriptome profiling of different tissues of *B. napus* revealed that *BnMLO2_2* had the most expression level in leaf and silique tissues among all the 7 *BnMLO2* members and also expressed higher in the SSR resistant accession than in the susceptible accession. In Arabidopsis, *mlo2* plants displayed reduced resistance to SSR, whereas overexpression of *MLO2* conferred plants an enhanced SSR resistance. Moreover, a higher expression level of *MLO2* showed a stronger SSR resistance in the transgenic plants. The regulation of *MLO2* in SSR resistance may be associated with the cell death. Collinearity and phylogenetic analysis revealed a large expansion of *MLO* family in *Brassica* crops.

**Conclusion:**

Our study revealed an important role of *BnMLO2* in the regulation of SSR resistance and provided a new gene candidate for future improvement of SSR resistance in *B. napus* and also new insights into understanding of *MLO* family evolution in *Brassica* crops.

**Supplementary Information:**

The online version contains supplementary material available at 10.1186/s13068-023-02325-z.

## Background

*Brassica napus* (*B. napus*) L. is the second most important oilseed crop following soybean (*Glycine max*) and world-widely cultivated for commercial uses such as human nutrition, animal feed, and energy resource [1–3]. *B. napus* (AACC, 2n = 4X = 38) is an allotetraploid species that originated from natural interspecific hybridization of two diploid species including *Brassica rapa* (*B. rapa,* AA, 2n = 2x = 20) and *Brassica oleracea* (*B. oleracea* CC, 2n = 2x = 18) [4]. It is well known as one of the most economically important oil crops in the world. Like the other field crops, *B. napus* also faced a severe threat of SSR disease caused by the necrotrophic fungus *Sclerotinia sclerotiorum* (*S. sclerotiorum*), especially in the humid environment, resulting in the significant reduction of seed yield and oil quality [5, 6]. In the United States, the losses caused by SSR have also exceeded $200 million every year [7]. Particularly in China, normally impact of SSR disease in terms of yield loss is between 10 and 20%, while severe attack (outbreaks season) may cause a reduction of seed yield up to 80% [8, 9]. The fungus *S. sclerotiorum* is identified as a necrotrophic fungus that can infect at least 400 different plant species, including *G. max* and *B. napus* [7, 10]. To date, there is no germplasm reported with a complete resistance to the fungal attack of *S. sclerotiorum*, and the  underlying defense mechanism of *B. napus* against *S. sclerotiorum* remains largely unknown. In the pathogen–plant interaction, when a plant perceives pathogen invasion, many genes respond rapidly to activate the immune responses. Just after the fungal attack, plants activate its primary defense system by activating pathogen-associated molecular patterns (PAMP)-triggered immunity (PTI), which enhance the expression of downstream genes, and then triggers a non-specific immune response [11, 12]. If pathogen attack is severe and can be able to break protective shield such as PTI, then secondary effector-triggered immunity (ETI) system will be activated by pathogen-specific avirulence effectors. These effectors can be recognized by host plant disease-resistance (*R*) gene coding proteins, which then activates the specific signal transduction pathways resulting in the enhanced expression of defense responsive genes [13, 14]. Subsequently, the hypersensitive responses (HR) are activated rapidly, and this resistance is long-lasting resistance [12, 15]. However, there is no *R* gene or resistance-related gene cloned in *B. napus* so far, which has shown the complete resistance against *S. sclerotiorum*. To date, breeding strategies in *B. napus* mainly relies on genotypic variations in germplasms with partial resistance. Earlier researches on resistance genes in *B. napus* are performed through quantitative trait loci (QTL) mapping, and they have successfully identified many SSR resistance QTLs, which are distributed on almost all the chromosomes except A4 on the A subgenome and C1 and C5 on C the subgenome [16–21]. With the rapid advancements in sequencing technologies, genome-wide association studies (GWAS) become a famous tool for gene-to-trait associations in many plant species. Application of GWAS is more robust, as it abandoned the lengthy process adopted for the construction of recombinant population, reprogrammed the phenotypic variation and genomic variation in the natural populations, and finally precisely predicted the genes or QTLs involved in complex traits based on linkage disequilibrium (LD) [22, 23]. Through the combinatorial approaches such as GWAS, transcriptome sequencing and biotechnological tools, numerous candidate genes conferring defense against *S. sclerotiorum* have been identified in *B. napus* [24–26]. Specifically, in susceptible (S) and resistant (R) cultivars of *B. napus*, some differentially expressed genes (DEGs) are identified at 48 h post-inoculation (hpi). These DEGs are involved in signal transduction, oxidative burst, bio-macromolecular transport, cell development, and biosynthesis of auxin, glucosinolate, jasmonic acid and ethylene [24, 25, 27, 28].

Powdery mildew (PM) is also a fungal disease and appeared world pread and severely damaged the legumes [29]. Its negative impact in terms of significant yield loss is also recorded in several other filed crops such as *Pisum sativum* [30]. The null allele of M*ildew Locus O* (*MLO*) was first reported in barley (*Hordeum vulgare*), which becomes famous for its significance against PM disease caused by the biotrophic ascomycete *Bgh* [31, 32]. In barley, natural and induced loss-of-function *mlo* alleles possess durable and broad-spectrum resistance to PM [32, 33], although the biological function and genetic mechanism behind the resistance conferred by most *mlo* genes have largely remained unknown. Owing to the high efficacy and robust crop improvement through utilization of *mlo* mutant gene, elite barley varieties with introgressed *mlo* alleles have already been cultivated in European Union since last four decades [31]. Though it is now established in barley, however, *mlo* also shows resistance in several other crop species like wheat, *Arabidopsis*, tomato, tobacco, pepper, and pea [30, 32, 34–40]. In detail, the *MLO* genes participate not only in the pathogen–host interactions, but also in several other physiological activities, for instance, *MLO* genes expression enhanced under rust infection in the resistant genotype of *Lathyrus sativus* varieties, which suggests that the *MLO* genes are also involved in rust resistance [41]. In rice, *OsMLO12* is involved in pollen hydration [42].

So far, a total 11–34 *MLO* genes have been identified in different plant species [43, 44]. For example, in *Arabidopsis* which belongs to a the *Brassicaceae* family, 15 homologous genes of barley *MLO*, and the functions of *AtMLO2*, *AtMLO6*, and *AtMLO12* were reported toassociate with PM disease [35]. As *Brassicaceae* is a large eudicot family, multiple rounds of different genome mergeing events appeared during its evolutionary history and were followed by genome duplications [45]. Likewise, polyploid *B. napus* genome was generated  through genome doubling following hybridization *B. napus* and *B. olerancea*. After polyploidization, the subgenomes undergo recurrent genomic rearrangements, resulting in gene loss and functional diversities. However, the genes located in the syntenic regions are rather conserved and can easily be identified [46–49]. Studying syntenic genes will be beneficial in learning the evolution in plants and it provides genetic material for crop improvement.

Several genes belonging to the *MLO* family have already been extensively studied in defense against biotrophic pathogens in different plant species. However, no research is currently available on *MLO* genes conferring resistance to the necrotrophic pathogens*.* In this study, we identified a *MLO* family gene *BnaC08g25340D* (*BnMLO2_2*) through GWAS that positively regulated the SSR resistance in *B. napus*. Moreover, previous and this study also showed that *MLO* is potentially associated with resistance to both biotrophic and necrotrophic pathogens. Analysis of SNP haplotypes, their phenotypic data from corresponding accessions and transgenic results in model plant *Arabidopsis* indicated that *AtMLO2* and *BnMLO2_2* were significantly correlated with SSR resistance. Besides, the expression pattern and evolution of the *MLO* gene family in *Brassicaceae* were analyzed. Our result revealed the detailed function of *BnMLO2_2* in SSR resistance and the asymmetrical evolution of *BnMLOs* gene in *B. napus.* The newly identified *BnMLO2_2* haplotypes or sequence variations will be potentially valuable for oilseed rape disease-resistance breeding. In a nutshell, our research provides new for genes regulating SSR and PM resistance in *B. napus * and new insights for the genetic improvement of polyploid crops.

## Results

### *BnMLO2_2* is associated with SSR resistance

**Fig. 1:**
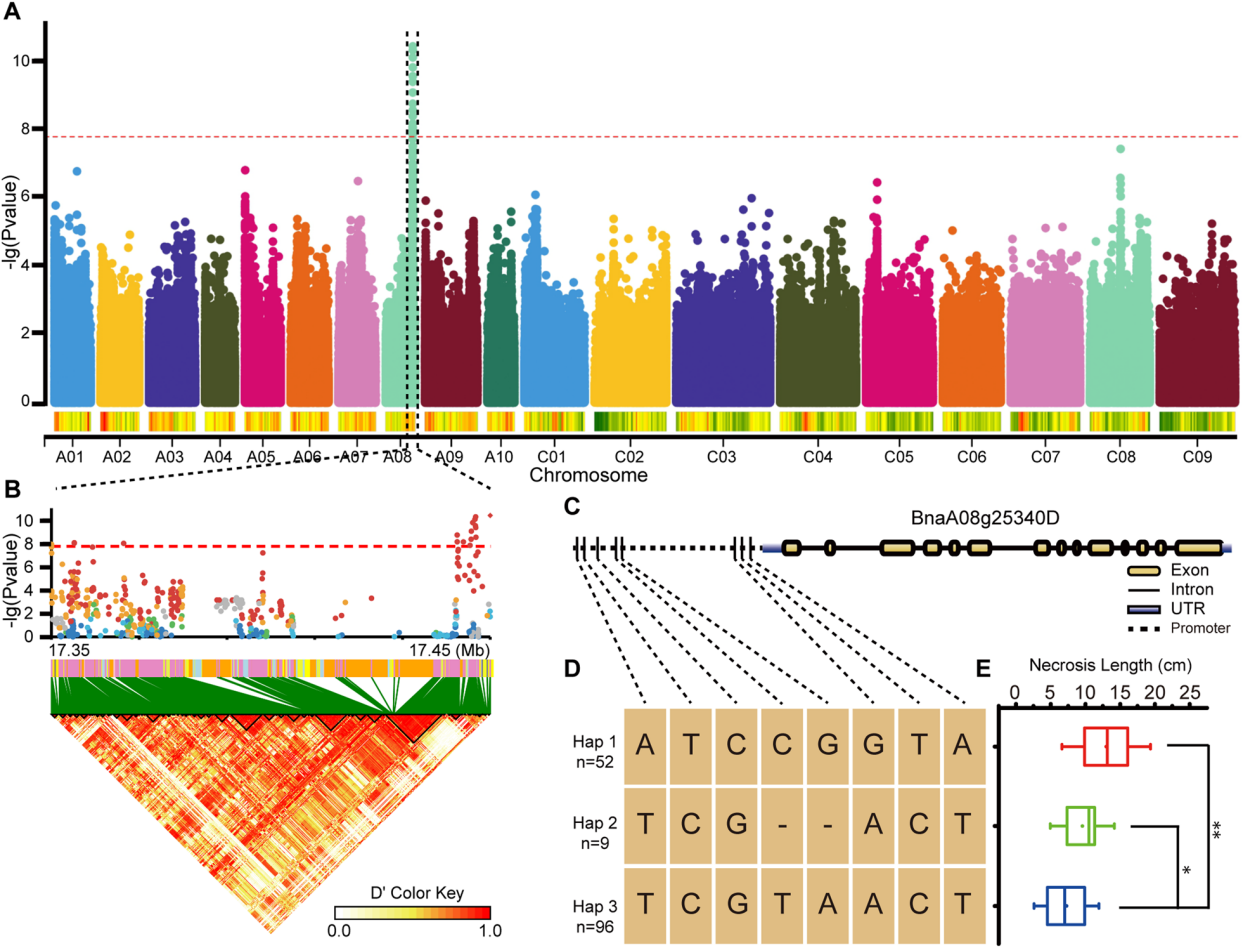
GWAS for SSR in *B. napus* and haplotypes analysis. **A**, Manhattan plot of GWAS. The dashed horizontal line means the Bonferroni-adjusted significance threshold (*P* = 1.799 × 10^–8^). **B**, Location of SNP loci associated with SSR resistance and pairwise LD between SNPs. SNP of *BnMLO2_2* in 17.35–17.45 Mb, the dots above the red dotted line denote the significantly associated SNPs. **C**, Gene structure and SNP variation in the promoter of *BnMLO2_2*. **D**, Haplotypes based on the SNPs combination in the promoter. **E**, The corresponding phenotypes of the three haplotypes

To detect loci regulating SSR resistance in the natural population, a GWAS was conducted in a panel of natural population of *B. napus* consisting of 222 accessions. Phenotypic measurements were defined as stem lesion length at 17 days post-inoculation (dpi) with *S. sclerotiorum* in the natural environment. The stem lesion length showed extreme variations as it ranges from 1.5 cm to 22.8 cm. A total of 2,779,265 SNPs were used to perform GWAS analysis using the general linear model. Our results showed that one significant block from 17.35 to 17.45 million base pairs (Mbs) on the chromosome A08 was strongly associated with SSR resistance (Fig. 1A and Additional file 4: Table S1). Among the 0.1-Mb block, the most significant SNPs were within the region of the *MLO* family gene, named *BnMLO2_2* (*BnaA08g25340D*), and the significant SNPs showed strong LD (Fig. 1B). To gain insight into the sequence variations in the region nearby *BnMLO2_2* coding sequence, SNPs in all accessions were thoroughly checked and then were classified into three haplotype groups based on the variant of eight SNPs. Each haplotype group contained at least 9 accessions (Fig. 1C). Similarly, in the corresponding phenotypic data, haplotype 3 was significantly different from haplotype 1 and haplotype 2, and accessions in haplotype 3 were more resistant to SSR (Fig. 1C and Additional file 5: Table S2). Therefore, we speculated that *BnMLO2_2* was associated with SSR resistance in *B. napus*.

### BnMLO2_2 positively regulates SSR resistance in B. napus

**Fig. 2:**
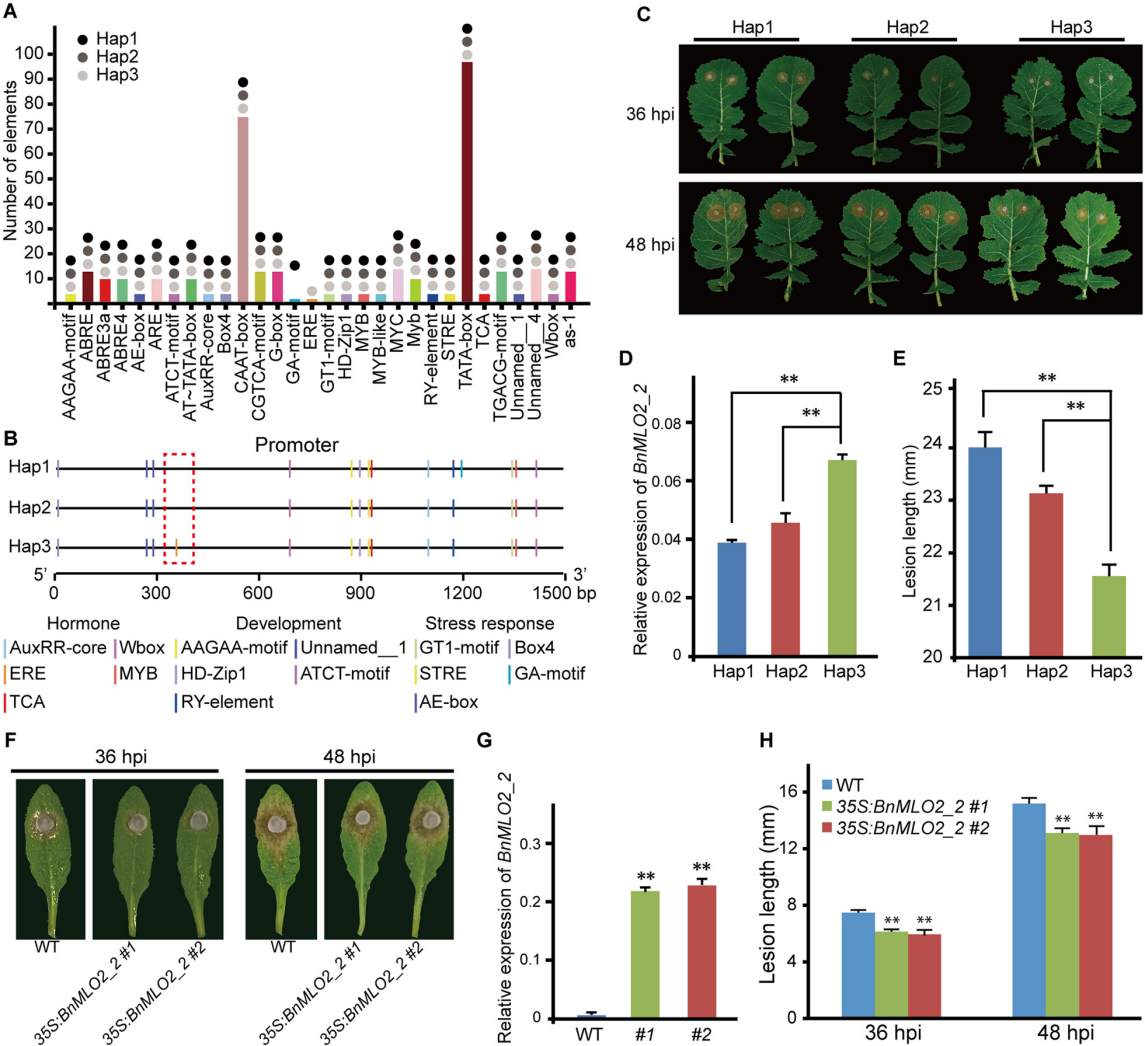
Cis-acting regulatory elements identification of *BnMLO2_2* and SSR resistance identification of B. napus. **A**, All cis-acting regulatory elements that were identified in three haplotypes. **B**, Single cis-acting regulatory elements and their location in the promoter. **C**, Phenotype of SSR resistance identification of the three haplotypes at 36 hpi and 48 hpi. **D**, The expression level of *BnMLO2_2* in accessions of the three haplotypes. **E**, Statistical analysis of phenotype at 48 hpi in the SSR resistance identification experiment. **F**, Phenotype of *Arabidopsis* transgenic plants and WT in the inoculation experiment at 36 hpi and 48 hpi. **G,** Statistical analysis of lesion length in the inoculation experiment of *Arabidopsis* transgenic plants and WT. **H**, Statistical analysis of phenotype in Arabidopsis WT and transgenic lines at 36 hpi and 48 hpi in the inoculation experiment. “**” means *p* < 0.05 in ANOVA test

Generally, promoter regions played a huge role in the regulation of gene expression at mRNA levels. As shown in Fig. 1, the *BnMLO2_2* promoter regions contain three types of variations distributed across our natural population, and among them, eight SNPs were highly associated with SSR resistance. Therefore, the cis-acting regulatory elements in the promoter regions of the three haplotypes were further investigated using related data retrieved from the “PlantCARE” database. Specifically, 1.5 kb upstream coding region of *BnMLO2_2* was analyzed, and a total of 30 cis-acting regulatory elements were obtained, and of these, 28 were found in three haplotypes (Fig. 2A, Additional file 6: Table S3), except GA-motif and ERE, which was only detected in accessions of haplotype 1 and 3, respectively (Fig. 2A, Additional file 6: Table S3). Moreover, the majority of cis-acting regulatory elements appeared multiple times across the population of each haplotype. For example, TATA-box appeared 32 times in each haplotype. Interestingly, 15 such single cis-acting regulatory elements were identified at least once in each haplotype (i.e., MYB and MTB-like). The above regulatory elements were categorized into three groups including development, hormonal and stress response. Besides, we also noted that, ERE was unique and appeared only in haplotype 3, and the GA-motif was unique and appeared only in haplotype 1 (Fig. 2B). Lastly, we analyzed the expression level of *BnMLO2_2* in the three haplotypes, and noted that expression level of *BnMLO2_2* in haplotype 3 was significantly higher than in the other two (Fig. 2D). So, we speculated that the unique cis-acting regulatory element ERE may be involved in the increment of higher expression level in haplotype 3. To test the stability of the three haplotypes, a leaf inoculation experiment was performed using the accessions of the three haplotypes. As expected, haplotype 3 showed less necrosis than haplotype 1 and haplotype 2 at both 36 hpi and 48 hpi (Fig. 2C). Additionally, the lesion length also showed a significant difference between haplotype 3 and the other two haplotypes (Fig. 2E, Additional file 7: Table S4). In summary, these results indicated that promoter difference led to the expression difference of *BnMLO2_2*, and this difference in the expression level of *BnMLO2_2* was associated with SSR resistance in *B. napus*.

To verify the function of *BnMLO2_2*, we cloned and overexpressed the *BnMLO2_2* of *B. napus* cultivar ZS11 (a resistant line from Hap3) into *Arabidopsis* and obtained two stable transgenic lines. Then, we performed the inoculation experiment with the leaves of *Arabidopsis* transgenic plants and WT. The leaves were inoculated with *S. sclerotiorum*. The results of inoculation experiment showed that the lesion length of transgenic plants in *35S:BnMLO2_2 #1* and *35S:BnMLO2_2 #2* was smaller than that of WT at 36 hpi and 48hpi (Fig. 2F), High expression of *BnMLO2_2* was detected in the transgenic plants (Fig. 2G). Statistical results also showed that there was a significant difference in lesion length between transgenic plants and WT (Fig. 2H). These results indicated that high expression of *BnMLO2_2* improved the resistance to resistance to SSR.

### Analysis of *BnMLO2* expression in various tissues of *B. napus* and responding to pathogen infection 

**Fig. 3:**
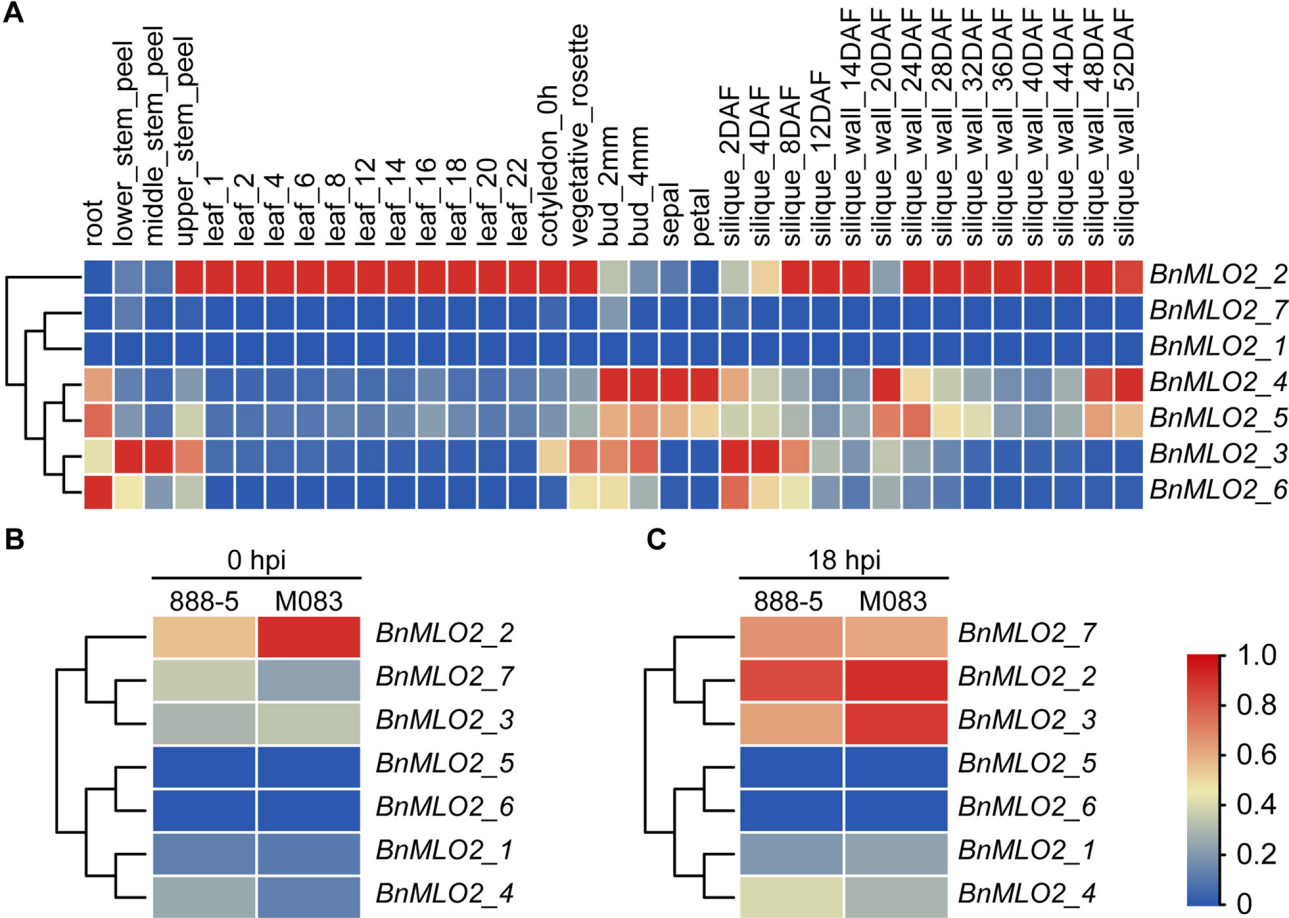
Expression pattern of *BnMLO2* members and their expression level in S and R cultivar lines. **A**, The expression level of 7 *BnMLO2* members in 35 tissues or stages of *B. napus* cultivar line ZS11 from BnTIR. **B**, The expression level of seven *BnMLO2* genes in 888-5 and M083 leaf before *S. sclerotiorum* inoculation, the expression of *BnMLO2_2* in R line was higher than that in S line. **C**, The expression level of seven *BnMLO2* genes in 888-5 and M083 leaf at 24 hpi with *S. sclerotiorum* inoculation, the expression of *BnMLO2_2* in R line was also higher than that in S line

Many genes expressed in different times and spaces and performed specific functions together to regulate the growth and development of plant organisms. There are seven *Arabidopsis* orthologous genes of *AtMLO2* reported in *B. napus* [50]. To understand their functions during the entire growth period, we used transcriptome data of 35 different tissues and stages of *B. napus* cultivar ZS11 retrieved from *B. napus* transcriptome information resource (BnTIR), a recently available database [51]. The fragments per kilobase of transcript per million mapped reads (FPKM) were used to evaluate the expression level of seven genes belonging to the *BnMLO* gene family. Results showed that, *BnMLO2_1* and *BnMLO2_7* were relatively expressed lower in all tissues, and *BnMLO2_2* was relatively higher expressed in leaf and silique tissues (Fig. 3A and Additional file 8: Table S5). Moreover, *BnMLO2_2* was a co-orthologs of the gene *AtMLO2*, which was involved in PM susceptibility [52, 53]. Thereby, *BnMLO2*s in the *BnMLO* gene family may be significantly associated with PM or other diseases in *B. napus*.

To investigate the expression difference of *BnMLO2* in the response to *S. sclerotiorum*, leaves of two *B. napus* cultivars, i.e., 888-5 (susceptible, S line) and M083 (resistant, R line) were inoculated. Both, control and under 18 hpi leaves of two cultivars were collected for RNA sequencing and then further analyses. FPKM value was used to evaluate the expression level of the seven members in control and treated leaves. Among the seven genes, *BnMLO2_5* and *BnMLO2_6* were not expressed in both lines, however, *BnMLO2_2*, *BnMLO2_3*, and *BnMLO2_7* genes were relatively expressed higher in both lines, and the expression of *BnMLO2_2* was the highest. Before *S. sclerotiorum* inoculation, *BnMLO2_2* was expressed higher in R line (Fig. 3B and Additional file 9: Table S6). After *S. sclerotiorum* inoculation, the expression of *BnMLO2_2* in R line was also higher than that in S line (Fig. 3C). The transcriptome data were later verified by using qPCR (Additional file 1: Figure S1). These results illustrated that SSR resistance may result from a high expression level of *BnMLO2_2*, and *BnMLO2_2* mediated SSR resistance may be inherent to the plants.

### MLO2 positively regulates SSR resistance in *Arabidopsis*

**Fig. 4:**
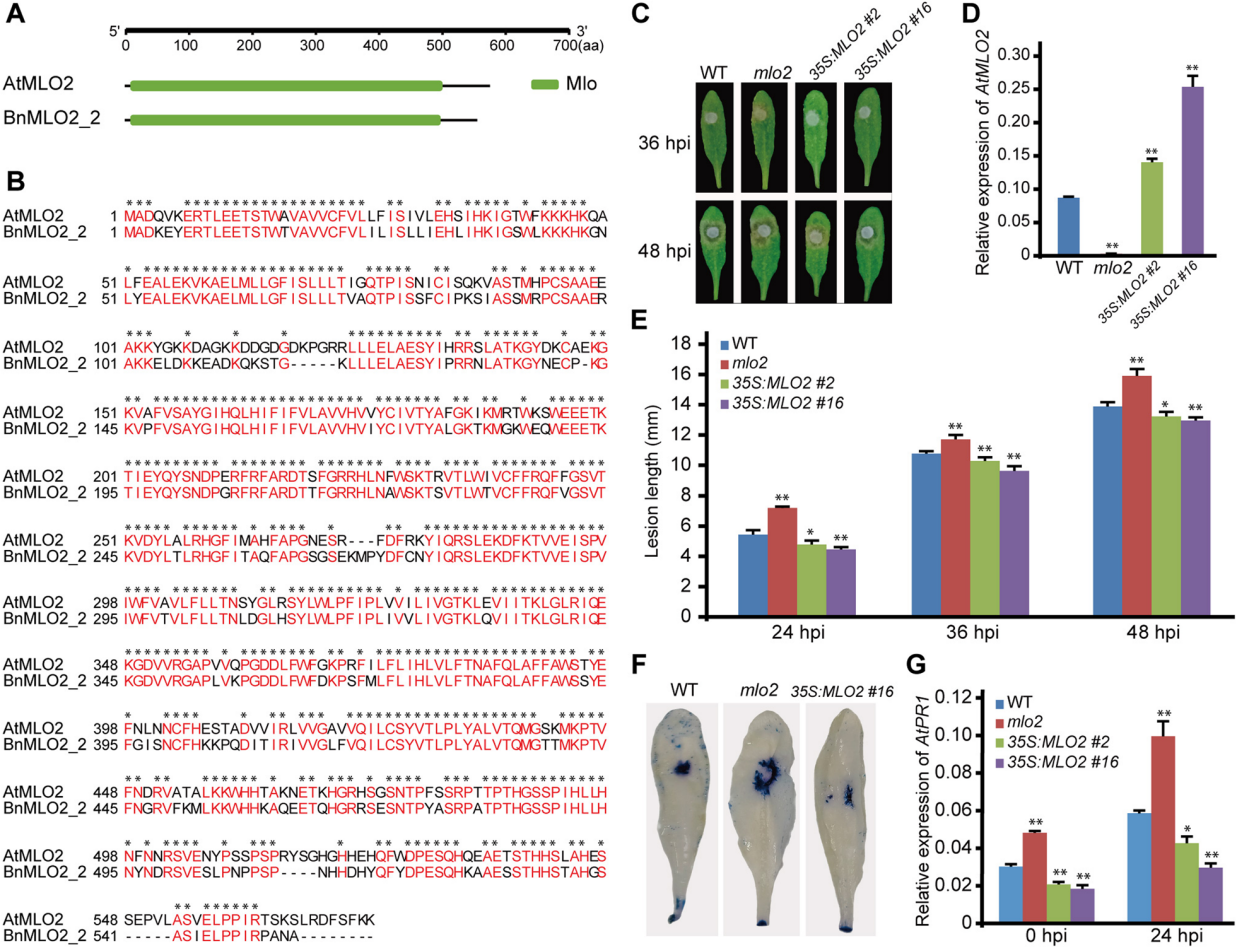
Sequence alignment and SSR resistance identification in Arabidopsis transgenic lines. **A**, Protein conserved domain of AtMLO2 and BnMLO2_2. **B**, Sequence alignment of BnMLO2_2 and AtMLO2, red letters indicate the same sequence. **C**, The phenotype of *Arabidopsis* WT, *mlo2*, and transgenic lines at different times after inoculation. **D**, Relative expression of *AtMLO2* in *Arabidopsis* WT, *mlo2*, and transgenic lines. **E**, Statistical analysis of phenotype in *Arabidopsis* WT, *mlo2*, and transgenic lines at 24 hpi, 36 hpi, and 48 hpi in the inoculation experiment. **F**, Cell death staining of *Arabidopsis* WT, *mlo2,* and transgenic lines at 36 hpi. **G**, Relative expression of *AtPR1* in *Arabidopsis* WT, *mlo2*, and transgenic lines at 0 hpi and 24 hpi. “*” means *p* < 0.05 in ANOVA test; “**” means *p* < 0.01 in ANOVA test

*AtMLO2* was an orthologous gene of *BnMLO2_2*, and their protein-conserved domains showed high similarity (Fig. 4A). Similarly, protein sequence analysis of AtMLO2 and BnMLO2_2 also showed high similarity, i.e., 73.12% (Fig. 4B). So, *AtMLO2* gene was speculated to be involved in positively regulating SSR resistance in *Arabidopsis*. A construct carrying *AtMLO2* coding sequence with 35S promoter was transformed into wild-type (WT) *Arabidopsis*. In the transgenic plants, the *AtMLO2* expression level was significantly increased compared to WT, and no expression was detected in the *mlo2* mutant plants (Fig. 4D). The WT, *mlo2*, and transgenic lines with *35S:MLO2 #2* and *35S:MLO2 #*16 were inoculated with *S. sclerotiorum* in the leaf, and the disease condition of *mlo2* and transgenic lines showed a significant difference in gene expression levels as compare with WT at 24 hpi, 36 hpi, and 48 hpi (Fig. 4D). Moreover, the lesion length of *mlo2* plants was longer than WT, and in transgenic lines lesion length was less than WT (Fig. 4E and Additional file 10: Table S7). These results indicated that the higher expression level of *AtMLO2* was positively related to SSR resistance in *Arabidopsis*. To further explore the resistance mechanism of *AtMLO2*, the leaves of 36 hpi were stained with trypan blue and observed cell death. As expected, in the transgenic plants exhibited less cell death than WT, and *mlo2* exhibited more cell death than WT (Fig. 4F). Additionally, the expression level of pathogenesis-related gene *PR1* was also checked, and results showed that the expression level of *PR1* was decreased in the transgenic plants and higher in *mlo2* plants as compared with WT before inoculation, however at 24 hpi, the expression was increased, but with no fold-change in the relative levels (Fig. 4G).

### Phylogenetic and syntenic relationship of *MLO* gene families in Brassica species

**Fig. 5:**
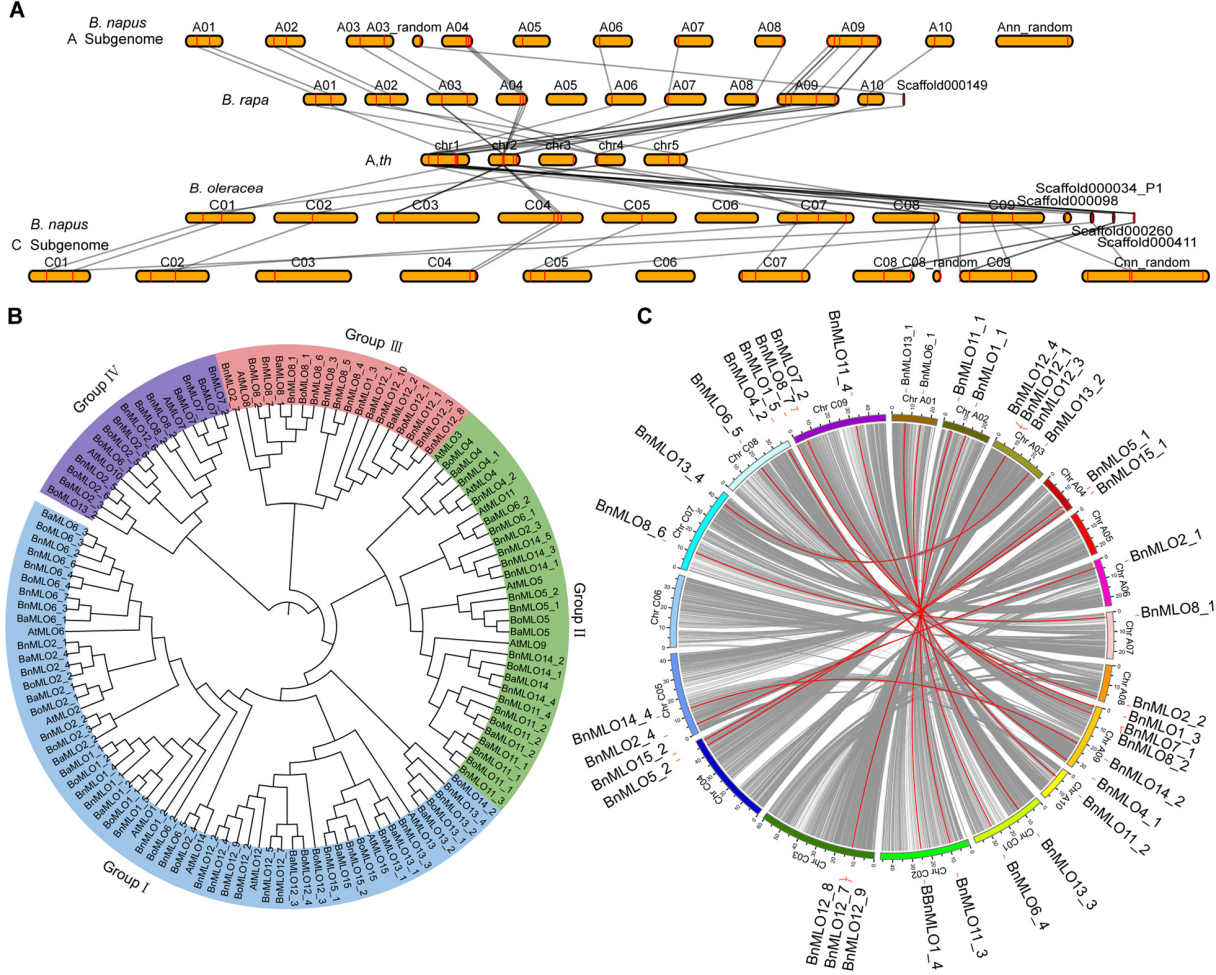
Evolution of *MLO* genes in Brassicaceae and the phylogenetic and syntenic relationship between A and C subgenome. **A**, The evolution of *MLOs* from *Arabidopsis to B. napus*. Orthologous gene identification was based on genomic alignments between *Arabidopsis*, *B. rapa*, *B. oleracea*, and *B. napus*. Black lines indicate the positional relationship of *MLO* genes. **B**, The phylogenetic relationship of *MLO* genes in Brassicaceae. A total of 123 protein sequences in *Arabidopsis*, *B. rapa*, *B. oleracea*, and *B. napus* were used to construct the phylogenetic relationship tree, different color represents different groups. **C**, Syntenic relationship between A and C subgenome. The circle consisted of different colors showing different chromosomes and the physical distance (Mb). Grey lines represent syntenic sequences, and the highlighted red lines indicated syntenic gene pairs of *BnMLOs*

*B. napus* was formed by the event of allopolyploidy between the recent ancestors of *B. rapa* and *B. oleracea*, which shared the ancient ancestor of *Arabidopsis* [55, 56]. During the brassicas genome duplications and genome mergers, *MLO* genes were embedded from *Arabidopsis* into the brassicas including *B. napus* (Fig. 5A). Using 15 *Arabidopsis* MLO protein sequences as query, 23, 28, and 57 genes were identified in *B. rapa* (*BaMLO*), *B. oleracea* (*BoMLO*) and *B. napus* (*BnMLO*), respectively, through the reciprocal BLASTP between the protein sequences retrieved from their respective genome databases (Additional file 11: Table S8). The 57 *BnMLO* genes were distributed across all the chromosomes of *B. napus*, and their gene length and physio-chemical properties were also different (Additional file 2: Figure S2, Additional file 12: Table S9). Among the *AtMLOs*, *AtMLO3*, *AtMLO9*, and *AtMLO10* genes have no orthologous genes in *B. rapa*, *B. oleracea*, and *B. napus*, however, *AtMLO12*, *AtMLO6*, and *AtMLO2* have the most of orthologous genes in *B. napus*. This replication model may imply their unusual biological functions. The *AtMLO* genes and their orthologous genes in *B. rapa*, *B. oleracea*, and *B. napus* were all used to analyze the evolution of *MLO* genes through phylogenetic tree analysis. A total of 123 MLO proteins were used to construct the phylogenetic tree (Fig. 5B). As shown in Fig. 5B, *MLO* gene families were classified into 4 groups, group I to group IV. Among, Group I was the largest group, which contained 57 *MLO* genes. The members with similar functions were classified into the same group, such as *AtMLO4* and *AtMLO11* were both associated with root morphogenesis and were classified in group II (Fig. 5B) [57, 58].

Many angiosperm species have experienced at least one round of whole-genome duplication (WGD) event through hybridization or subsequent chromosome doubling, this phenomenon is called a polyploidization event [47]. In the case of *B. napus*, the gene function divergence in different subgenomes is becoming more significant. So, the enormous syntenic blocks between A and C subgenomes were identified based on aligned syntenic chromosomal regions. Each chromosome had several syntenic blocks in the corresponding A and C subgenome, but not all the genes or blocks had a syntenic region (Fig. 5C). In total, 24 syntenic gene pairs of *BnMLO* were identified with a higher identity according to their reference protein sequences, however, 5 pairs of *BnMLO* syntenic gene were mapped on the unknown chromosomal regions, so they were not displayed (Fig. 5C). The other 9 *BnMLO* genes were also not shown in the figure because none of the syntenic genes was found between the A and C subgenomes (Fig. 5C). Owing to the complex genomic structure of hetero-tetraploid and the agricultural importance of oilseed rape, *B. napus* is becoming a model species for the research of evolutionary consequences following polyploidy (Additional file 13).

### Gene structure and conserved domains of *BnMLO* genes

**Fig. 6:**
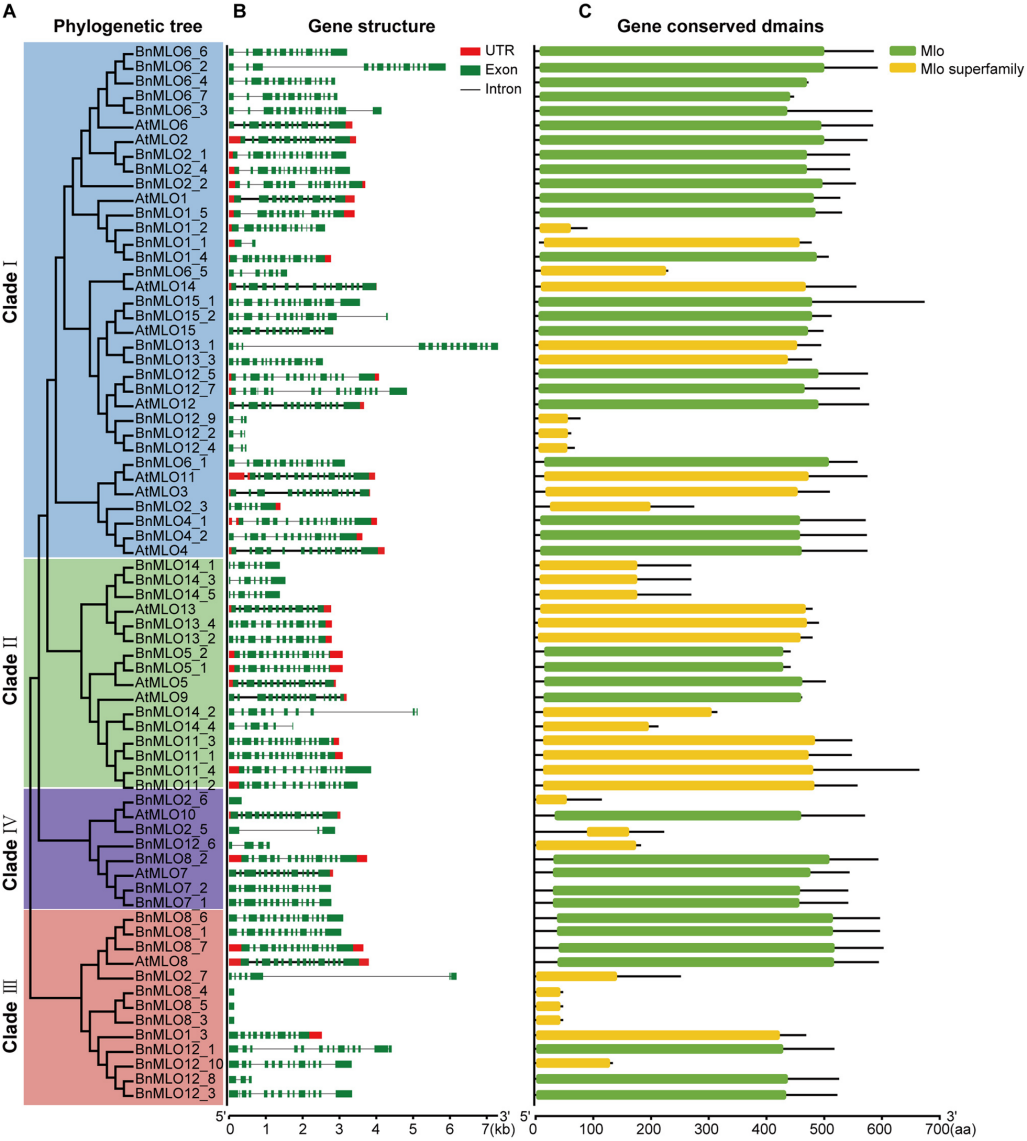
Gene structure and conserved domains of *BnMLO* genes. **A**, The phylogenetic tree of AtMLO and BnMLO proteins, in which four clades were classified. **B**, Gene structure of 15 *AtMLO* and 57 *BnMLO* genes. The untranslated regions (UTR) and exons are indicated by red and green boxes, respectively, the introns are shown with black lines. **C**, Gene conserved domains of 15 AtMLO and 57 BnMLO proteins. MLO and MLO family conserved domains are represented in green and yellow boxes. The scale label is displayed at the bottom of the figure. kb: kilobase pairs; aa: amino acid

To analyze the gene structure and conserved domains of the 15 *AtMLO* and 57 *BnMLO* genes, a new phylogenetic tree was constructed containing four classified clades (Fig. 6A). The genes with high sequence similarity were clustered together. A wide difference was recorded in the transcript length of the 15 *AtMLO* and 57 *BnMLO* genes with a minimum of 141 base pair (bp) and a maximum of 7,303 bp, and the peptide length ranged from 46 to 672 amino acid (aa), and the number of exons varied from 1 to 16 (Fig. 6B and Additional file 8: Table S5). In which, *BnMLO12_2*, *BnMLO8_3*, *BnMLO8_4*, and *BnMLO8_5* consisted of only one exon, and with no untranslated region (UTR) either, but 40.4% (23/57) of the genes contained UTR region either in the upstream or downstream of the coding sequence. For some genes, such as *BnMLO2_7*, and *BnMLO13_1* the exons were extended by a large intron. Some of the syntenic genes, for example, *BnMLO5_1* and *BnMLO5_2* have similar structures, however, *BnMLO14_2* and *BnMLO14_4* were significantly different (Fig. 6B). Although the protein length of the *BnMLO* genes was varied, however, all 57 proteins contained the MLO domain or MLO superfamily domain, and no other conserved domain was detected (Fig. 6C). Most of the MLO conserved domains were distributed in the N-terminal, and some of the proteins only contains a MLO conserved domain, such as *BnMLO12_6*, and *BnMLO12_10* (Fig. 6C).

Because of the co-existence of short and long fragments in *BnMLO* genes as well as the large intron inside the exons of several genes (such as *BnMLO12_7* and *BnMLO13_1*), it is obvious that a small-scale duplication, a large fragment missing and transposons insertion occurred during the evolution. So, the transcriptional efficiency as well as biological function may be altered.

## Discussion

### *BnMLO2* is salient in the *BnMLO* gene family

In this study, we identified a *BnMLO* gene that was associated with SSR resistance in *B. napus*, the *BnMLO2_2* was the orthologues gene of *AtMLO2*. *AtMLO2* positively regulated the SSR resistance in *Arabidopsis*, SNP haplotypes of *BnMLO2_2* were also associated with SSR in the natural population of *B. napus*. We analyzed another published transcriptome data of 12 different tissues of ZS11 and found that, among the 57 *BnMLO* genes, except *BnMLO2*, other *BnMLO* genes were relatively lowly expressed (Additional file 3: Figure S3) [59]. So, the high expression level of *BnMLO2_2* may take primary responsibility in response to SSR resistance. Other *BnMLO* genes were not detected to be associated with SSR resistance through GWAS analysis. However, some other homologous genes of them were reported and potentially to be involved in the growth and development [42, 58, 60, 61]. These *MLO* genes developed function divergence during evolution. According to the inoculation experiment, *BnMLO2_2* was highly expressed before and after inoculation in both resistant and susceptible cultivar lines, and differences appeared between cultivar lines. Therefore, the *BnMLO2_2* mediated resistance for *S. sclerotiorum* was nonspecific. In *Arabidopsis*, *AtMLO2*, *AtMLO6* and *AtMLO12* represented the co-orthologs of barley *MLO* [62], and *mlo2* mutant that also showed high resistance to PM [35], these findings indicated that *mlo*-based disease resistance is not only restricted to barley and can be used for other commercial crops including *B. napus*.

### Role of cell death in combating with biotrophic and necrotrophic pathogens

In barley, *mlo* possesses durable broad-spectrum resistance to PM [33], similarly in some other species like tomato, tobacco, and pepper, *mlo* also showed resistance to PM, and was considered to be a resistant alleles. However, in our research *Atmlo2* mutant showed more susceptibility to *S. sclerotiorum*, and the overexpression of *AtMLO2* enhanced the resistance to *S. sclerotiorum*. This different result was due to the *S. sclerotiorum* being a necrotrophic pathogen. Moreover, the *Atmlo2* mutant caused spontaneous cell death and usually represented early senescence in the rosette leaves [63]. Different from PTI, plant ETI aroused programmed plant-host cell death (PCD) at the microbial invasion sites. ETI is coevolved and highly specialized and is also considered the major disease resistance method for biotrophic and hemi-biotrophic pathogens [15]. In the present research, *Bgh* belongs to the biotrophic fungal pathogens, PTI and ETI are both used to prevent pathogen diffusion, especially in ETI reaction and hypersensitive response (HR)–associated cell death, which conferring pathogen growth by abolishing its nutrient supply. However, *S. Sclerotiorum* belongs to necrotic fungus, and cell death occurs during early infection of necrotrophs, which is an indication of a successful invasion. Further activation of cell death immensely enhances the colonization of necrotrophic pathogens [64–66]. The dead cells provided abundant nutrients for the growth of necrotrophic pathogens. So, the *Atmlo2* mutant was more susceptible to *S. Sclerotiorum*. On the other, *AtMLO2* and *BnMLO2_2* were positively involved in regulating SSR resistance. As shown in Fig. 4, the transgenic-positive plants exhibited less cell death than WT, *mlo2* exhibited more cell death than WT, and the *MLO2*-mediated SSR resistance was achieved by inhibition of cell death.

Plant resistance to *S. sclerotiorum* has been known to be quantitative and the underlying mechanisms are complicated. *PR1* possesses antimicrobial activity and its overexpression confers plants an enhanced Sclerotinia resistance in some literature. However, *PR1* was also used as a marker gene for plant cell death [67]. *S. sclerotiorum* is a necrotrophic fungal pathogen and could take advantage of plant cell death for its survival. In our study, the *mlo* mutant displayed spontaneous cell death which may play a major role in plant susceptibility to *S. sclerotiorum* although *PR1* expression was highly induced. In fact, some *Arabidopsis* mutants with higher *PR1* expression but decreased resistance to necrotrophic pathogens were also reported like *adc* (arginine decarboxylase enzymes) mutants [68]. This was consistent with our results. In our study, high *AtPR1* expression in the mutant was not inconsistent with poor resistance.

### Asymmetrical evolution of *B. napus*

The consequences of WGD following polyploidy have occurred in the form of gene diploidization, and gene loss has always been considered an important evolutionary energy in many organisms [69, 70]. Polyploids are mainly formed by the hybridization of two species and subsequently chromosome doubling. So, several genes existed with multiple copies that belong to specific gene families and are responsive to potential biotic and abiotic stresses during environmental adaptation. The diversification that appeared in the multi-copy genes either due to small-scale duplication or deletions often come with some sort of useful novelties [71]. For instance, research focused on the potential impact of transcription factors (TFs) in some metazoans resulting from WGDs showed that obtaining a novel function of TFs required a long time stabilized retention of insertion after WGDs [72]. However, during the origination and evolution/speciation of polyploids, even gene diploidization and gene loss are largely unknown. In the present study, *MLO* genes were identified in *B. napus*, *B. rapa* and *B. oleracea*, respectively, there were 23 *BraMLO* genes, 28 *BolMLO* genes, and 57 *BnaMLO* genes were found. The *BnaMLOs* originate from *BraMLOs* and *BolMLOs*, however, the numbers of *BnaMLOs* are not in accord with the summation of *B. rapa* and *B. oleracea* (Additional file 11: Table S8). This probably occurred in *B. napus* due to the several consequences following polyploidy, such as the inhomogeneous distribution of chromosomes, different gene lengths, gene structure, genome rearrangement, gene duplication, large fragment missing, transposons insertion, and gene loss. The above findings provided the theoretical basis for the functional divergence in *MLOs*. However, a better understanding of WGD following polyploidy will help us to face challenges in agriculture and crop improvement. Excited about the high efficacy and robust crop improvement in barley, we are sure that *mlo* genes or alleles can be used in other crop species including *B. napus*.

### Function divergence in the *MLO* gene family

It is generally considered that polyploids have stronger mutation robustness than diploids, which results in an increased potential for environmental adaptation during environmental turmoil [73, 74]. This may partially be attributed to the gene function divergence of duplicate genes. In *Arabidopsis* and barley, *mlo* mutants often exhibited abnormal growth, such as spontaneous cell death, callose deposition, and early senescence [35, 75, 76]. Moreover, *MLO* was found to be involved in different cellular processes in growth and development. In our research, gene function divergence also existed in the 57 *BnMLO* genes as they showed different expression levels in different tissues under natural conditions. As shown in Fig. 3, *BnMLO2_6* was highly expressed in the root, *BnMLO2_3* was highly expressed in the stem, and *BnMLO2_4* was highly expressed in the bud, sepal, and petal. Therefore, during the whole growing period, *BnMLOs* may be directly or indirectly involved in the developmental period of an organism—including root morphogenesis and perception of normal pollen tubes. Besides developmental cues, *BnMLOs* also showed resistance against biotic and abiotic stresses [58, 60, 61]. However, the expressions of members of *BnMLO7* and *BnMLO14* genes failed to be detected in all the 12 tissues of *B. napus*, indicating their function differentiation. The divergence in *BnMLO* genes during polyploidization may take responsibility for the functional diversity.

## Conclusions

Here, we used a natural population of *B. napus* consisting of 222 accessions to perform GWAS analysis targeting SSR resistance traits. Our results revealed that *BnMLO2_2*, a member of seven homolog genes of *Arabidopsis MLO2*, was closely associated with SSR resistance. The *MLO* gene was first identified as associated with resistance to both biotrophic and necrotrophic pathogens. Moreover, three haplotypes were identified based on SNP variations in the promoter region of *BnMLO2_2*, which was found to be involved in the changes of the expression levels of the *BnMLO2_2* in the leaves and silique tissues and were responsible for the SSR resistance variations observed in the population used in this study. Overexpression of *AtMLO2* resulted in SSR resistance in *Arabidopsis*. The mechanism underlying *MLO2* in the regulation of SSR resistance was associated with cell death and was inherent. Syntenic analysis revealed the asymmetrical evolution between the A and C subgenomes of *B. napus*. This is the first study on the roles of *MLO2* in the regulation of SSR resistance, and the results show that the genetic modifications of *BnMLO2_2* are promising for the improvement of SSR resistance in *B. napus*.

## Materials and methods

### SNP and GWAS

The resequencing data were based on published data from our laboratory [77]. GWAS was performed in R script under the general linear model (GLM) using 2779265 SNPs [78]. The minor allele frequency (MAF) ≥ 0.05. The significance threshold for GWAS was set to *P* < 1.799 × 10^–8^ (0.05/SNP number). The Manhattan plot was displayed through qqman software [79].

### Plant materials and fungal pathogen materials

The materials used for GWAS were collected from all over the world, including spring, winter, and semi-winter ecotype accessions, and were cultivated under natural growing conditions. The phenotype data were collected in 2019. *B. napus* cultivar line ZS11, 888-5, M083, and the 222 *B. napus* natural varieties all came from our laboratory and were planted in our experimental field in Yangluo, Wuhan, Hubei province, China. Every year the natural varieties were planted in three replicates and developed by self-pollination; all the lines had been purified for many years. The *S. sclerotiorum* strain 1980 was preserved in our laboratory, and the mycelia were cultured on potato dextrose agar medium at 24 °C in the dark. The advancing edge of growing mycelia was selected to make the mycelia plug for inoculation.

### Stem inoculation with *S. sclerotiorum*

The 222 *B. napus* natural varieties were planted in the experimental field in October, plants grow normally. On March, 10 individual plants of the 45 plants in each plot were inoculated with the *S. sclerotiorum* at the same place in the stem of each plant using special mycelium plugs [80]. For each plant, the stem was inoculated with an 8-mm mycelial plug and fixed with plastic film. After inoculation, the plants grew normally in the natural environment. The inoculation experiment was performed with three replicates under the same environment. We measured the lesion length at 17 dpi, the phenotype used for GWAS was the average value of the three replicates.

### Leaf inoculation with S. sclerotiorum

For *B. napus* leaf, both sides of the leaf vein were inoculated with two mycelia plugs 8 mm in diameter. For the *Arabidopsis* leaf, the leaf vein was inoculated with a mycelial plug of 6 mm in diameter. After inoculation, the leaves were put into boxes to retain humidity, subsequently, the boxes were put in the dark under 24 °C. The inoculation experiment was performed with three replicates under the same environment. We measured the length of two vertical diameters at 24 hpi, 36 hpi, 48 hpi, respectively, and the lesion length of a single leaf was presented by the average value of two diameters, the lesion length was presented by the average of two diameters, The phenotype of one *B. napus* haplotype or *Arabidopsis* line was presented by the mean value of the three replicates. In the SSR resistance identification experiment of *B. napus*, leaves of haplotype 1 and haplotype 3 were collected from 15 accessions, leaves of haplotype 2 were collected from 8 accessions, and each haplotype included three biological repeats.

### Identification of *BaMLOs*, *BoMLOs*, and *BnMLO*s

Sequences of the 15 AtMLO proteins were downloaded from the *Arabidopsis* genome database (https://www.arabidopsis.org/) and were used as queries for the identification of *MLO* genes in *B. rapa*, *B. oleracea*, and *B. napus*. The genome information of *B. napus*, *B. rapa*, and *B. oleracea* was obtained from the BRAD (Brassicaceae Database) database (http://brassicadb.cn/#/) [50]. The HMMER3.0 (http://www.hmmer.org/) was used to search for MLO genes (E value was set to 1e-5) [81].

### Overexpression of *AtMLO2*

The *AtMLO2* coding sequence was cloned from *Arabidopsis* WT, with the forward primer MLO2_F (5′-GAGAACACGGGGGACTCTAGAATGGCAGATCAAGTAAAAG-3′) and the reverse primer MLO2_R (5′-GTGGTGGTGGTGGTGGGTACCTTTCTTAAAAGAAAAATC-3′). The *BnMLO2_2* coding sequence was cloned from *B. napus* cultivar ZS11, with the forward primer MLO2_2_F (5′- TTGGAGAGAACACGGGGGATGGCGGATAAGGAATATGAGAG-3′) and the reverse primer MLO2_2_R (5′-ACTAGTCAGATCTACCATAGCGTTTGCTGGTCGTATAGG-3′). The vector pBI121 was digested with restriction endonucleases *Kpn I* and *Xba I*, then recombined with the *BnMLO2_2* and *AtMLO2* coding sequence through homologous recombination in vitro. Then transformed the construct into *Escherichia coli* for sequencing. Selecting the correct monoclonal and then transforming it into agrobacterium and *Arabidopsis*. The T_2_ transgenic-positive plants and WT were used for the inoculation experiment.

### Establishment of phylogenetic relationship

The initial phylogenetic relationship tree was established through MLO protein sequences from *Arabidopsis*, *B. rapa*, *B. oleracea*, and *B. napus*. Phylogenetic trees were generated with MEGA11 software using the Neighbor-Joining (NJ) method with default settings. The phylogenetic relationship tree then was modified using EvolView, an online service tool (https://www.evolgenius.info/evolview/#login).

### Analysis of *BnMLO* gene structure and protein conserved domains

The BnMLO protein sequences were used to investigate conserved domains through the Batch search provided by Pfam (http://pfam.xfam.org/search#tabview=tab1) online tool and then investigated via TBtools [82], all the parameters were used as displayed in. The information of *BnMLO* exon and intron structures were extracted with generic feature format (GFF) files and were downloaded from the BRAD (http://brassicadb.cn/#/). The gene structures were established through GSDS2.0 (http://gsds.cbi.pku.edu.cn/index.php), an online service tool for gene feature visualization.

### Transcriptome analysis in *B. napus* cultivar lines

Transcriptome data of 35 different tissues or stages from *B. napus* cultivar line ZS11 were obtained from BnTIR online database (http://yanglaboratory.hzau.edu.cn/BnTIR). Transcriptome data of 12 tissues of ZS11 were obtained from the National Center for Biotechnology Information (NCBI) (SRA accession: PRJNA474576) [59]. Transcriptome data of 888-5 and M083 were not published. Clean reads were mapped onto the reference genome of *B. napus* ‘Darmor-bzh’ [55]. Heatmap was constructed using FPKM values through TBtools.

### RNA extraction and quantitative real-time PCR analysis

qPCR was performed to verify the RNA-seq result. The total RNA of leaf samples was extracted using TRIzol reagent (Invitrogen, Carlsbad, CA, USA). 2 μg of RNA was used to reverse transcribe cDNA using the PrimeScriptTM RT reagent Kit with gDNA Eraser (TaKaRa Co., LTD, Beijing, China). The *B. napus* β-actin gene (AF111812) was used as a reference standard. The relative expression level was calculated using the 2^−△△^Ct method [83]. In the SSR resistance identification experiment of *B. napus*, the RNA of each haplotype was extracted from a mixture of five accessions, and each RNA included three biological repeats. Primer sequences used in this study have been provided (Additional file 13:Table S10).

## Supplementary Information

**Additional file 1: Figure S1.** Histogram of gene relative expression level obtained by qPCR and the correlation analysis between qPCR and RNA-Seq.

**Additional file 2: Figure S2.** Chromosomal location of 57 *BnMLO* genes.

**Additional file 3: Figure S3.** The expression level of 57 BnMLOs in 12tissues of ZS11.

**Additional file 4: Table S1.** GWAS result of A08 chromosome.

**Additional file 5: Table S2.** The phenotype of accessions in each haplotype, and the statistical analysis. 888-5 and M083 before and after inoculation.

**Additional file 6: Table S3.** Predictive analysis results of cis-acting regulatory elements for the three haplotypes.

**Additional file 7: Table S4. **Phenotype statistical analysis of each haplotype in leaf inoculation experiment.

**Additional file 8: Table S5.** FPKM value of *BnMLO2* gene expression in different tissues and stages of ZS11.

**Additional file 9: Table S6.** FPKM value of *BnMLO2* gene expression in 888-5 and M083 at o hpi and 18 hpi.

**Additional file 10: Table S7.** Statistical analysis of lesion length in WT, *mlo2* and transgenic lines at 24 hpi, 36 hpi, and 48 hpi.

**Additional file 11: Table S8.** Orthology of *MLO* genes in *Arabidopsis*, *B. rapa*, *B. oleracea* and *B. napus*.

**Additional file 12: Table S9.** Gene location and protein Characteristics of *BnMLOs*.

**Additional file 13: Table S10.** Primers used in this study.

## Data Availability

The referenced data can be obtained from the corresponding article and NCBI. Some of the original data in this study are represented in the supplementary material, and some data are being published.
