# Supplementary figures and images for: Functional and evolutionary study of MLO gene family in the regulation of Sclerotinia stem rot resistance in Brassica napus L
Source: Biotechnol Biofuels Bioprod. 2023 May 23;16:86. doi: 10.1186/s13068-023-02325-z (PMC10204302; doi:10.1186/s13068-023-02325-z)

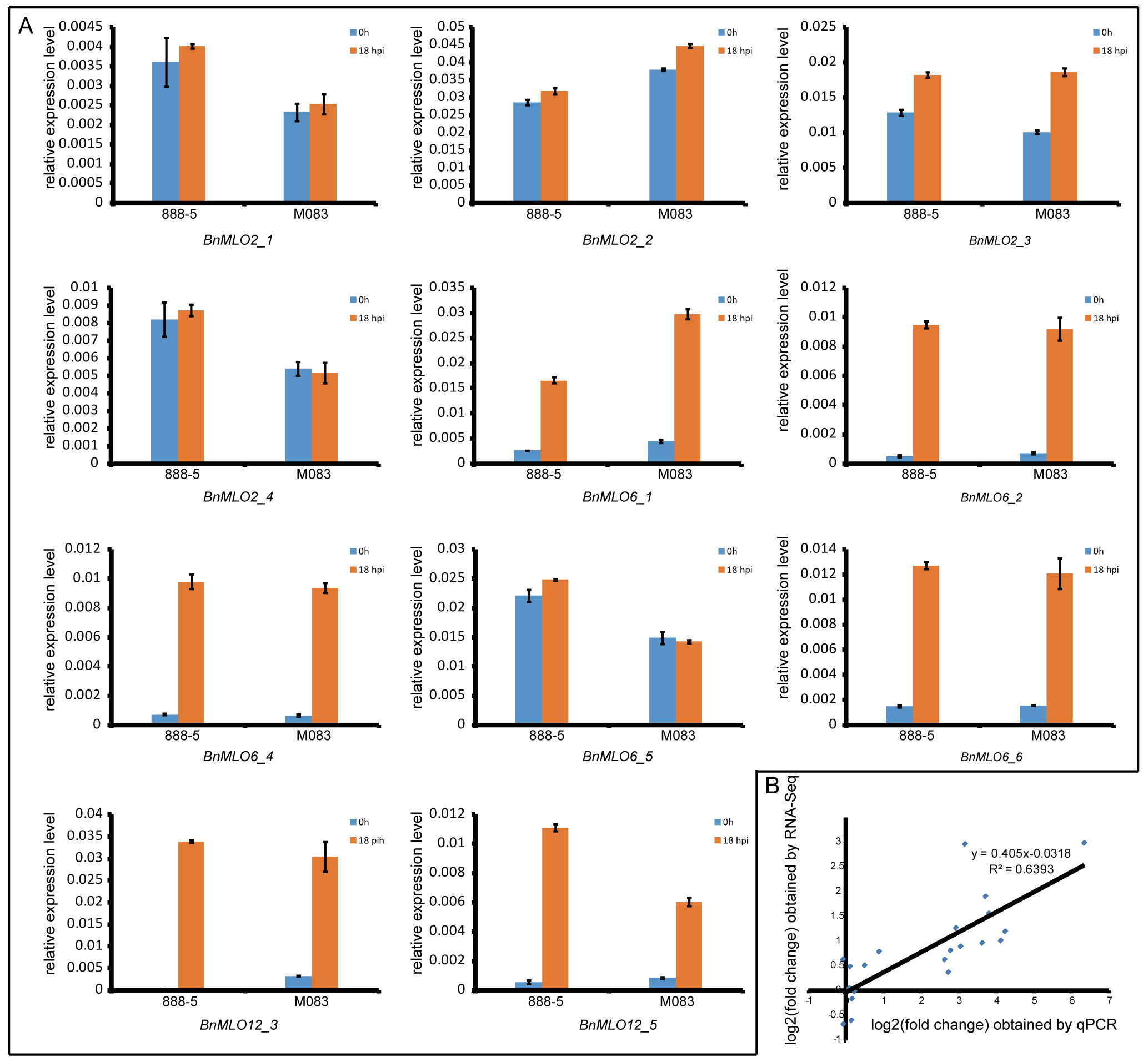

Supplement: Supplementary file 1 — Additional file 1: Figure S1. Histogram of gene relative expression level obtained by qPCR and the correlation analysis between qPCR and RNA-Seq. [file 13068_2023_2325_MOESM1_ESM.tif]

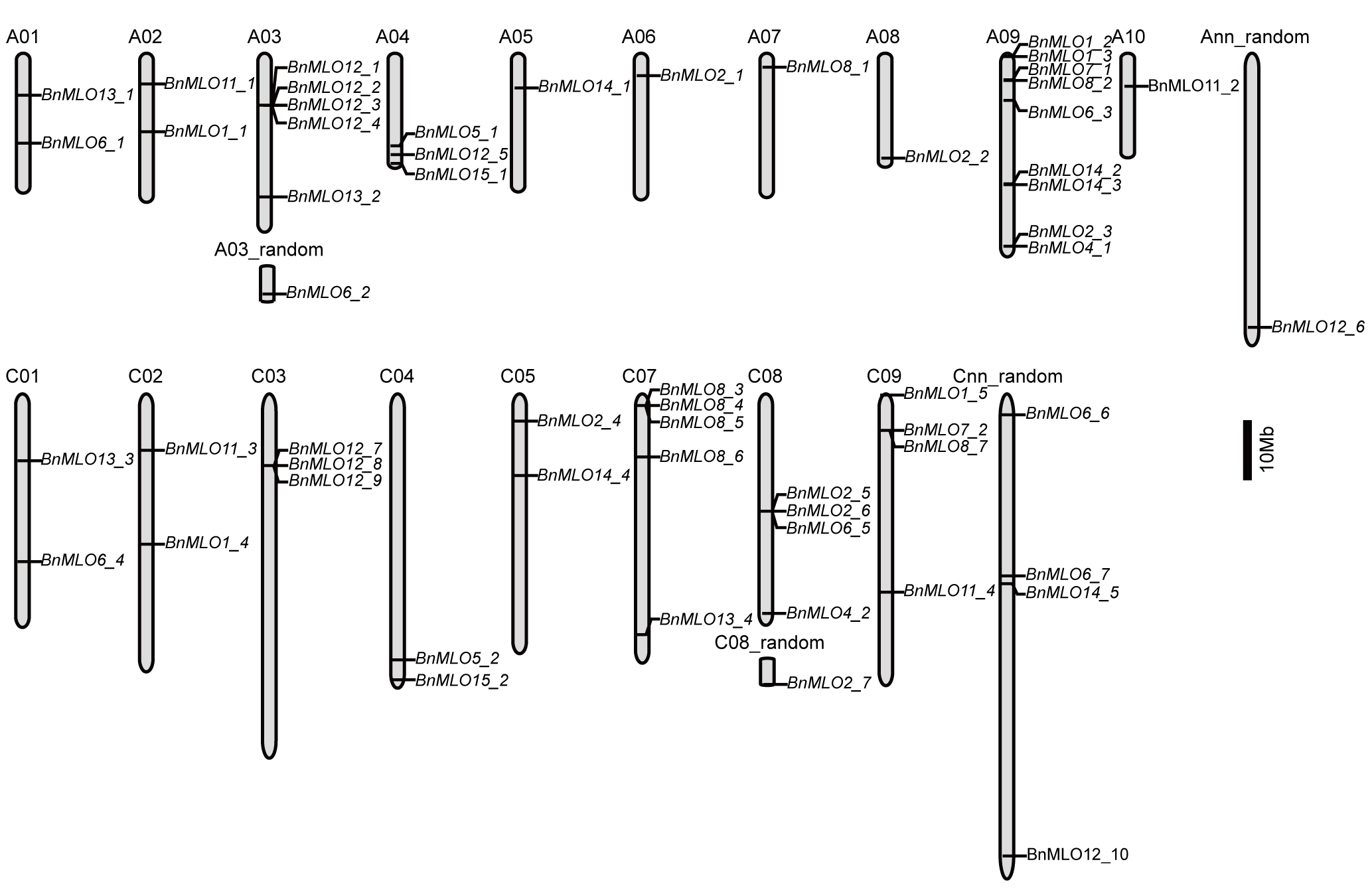

Supplement: Supplementary file 2 — Additional file 2: Figure S2. Chromosomal location of 57 BnMLO genes. [file 13068_2023_2325_MOESM2_ESM.tif]

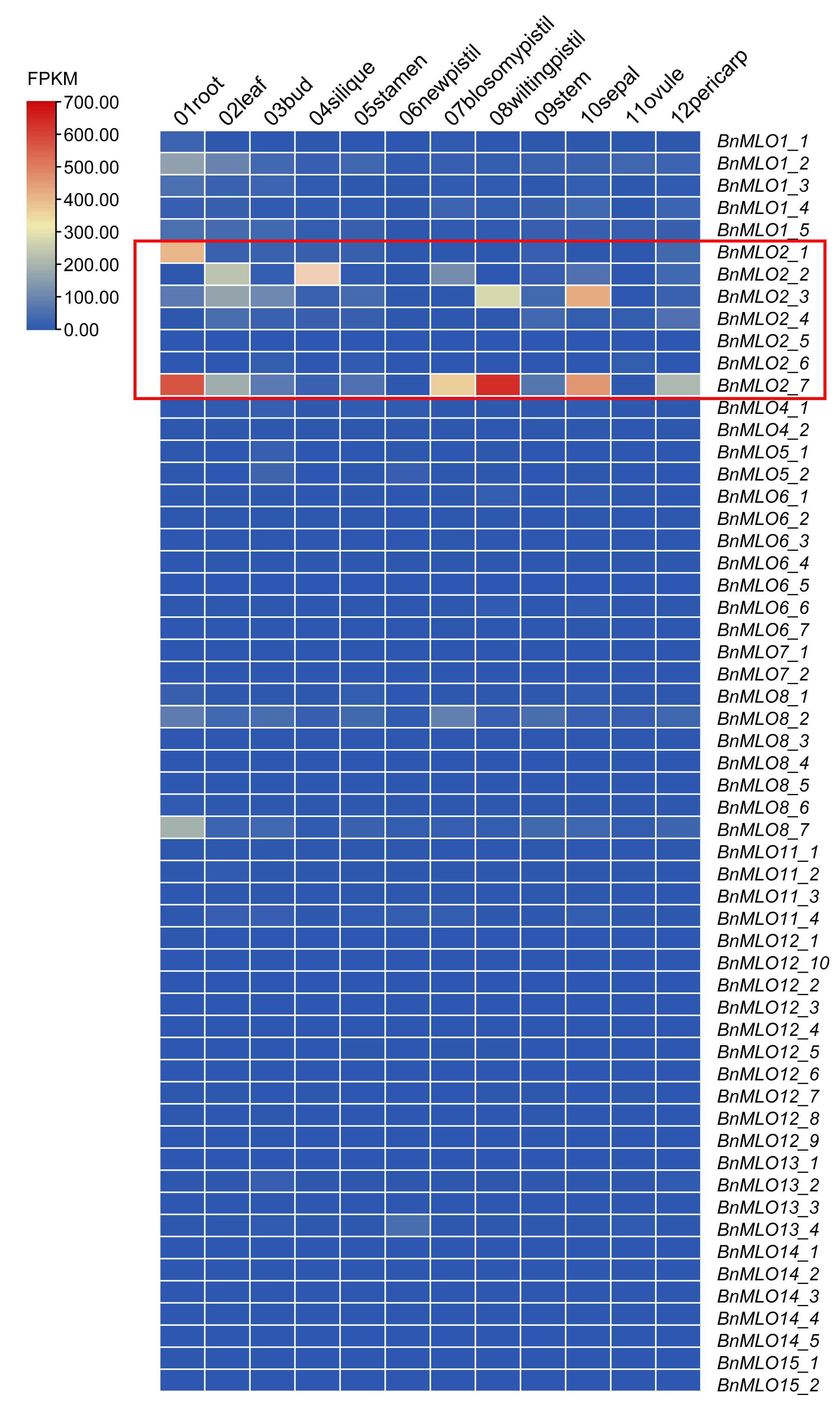

Supplement: Supplementary file 3 — Additional file 3: Figure S3. The expression level of 57 BnMLOs in 12tissues of ZS11. [file 13068_2023_2325_MOESM3_ESM.jpg]
